# Isolated Hepatic Artery Dissection in a Nonsurgical Patient: A Case Report and Literature Review

**DOI:** 10.7759/cureus.104729

**Published:** 2026-03-05

**Authors:** Aashima Gupta, Thomas Ruta, Claire Kissinger, Mina Aiad, Melissa Wilson

**Affiliations:** 1 Internal Medicine, St. Luke's Hospital, Bethlehem, USA; 2 Hematology and Medical Oncology, St. Luke's University Health Network, Easton, USA; 3 Hematology and Medical Oncology, St. Luke's University Health Network, Bethlehem, USA; 4 Hematology and Medical Oncology, St. Luke's Hospital, Bethlehem, USA

**Keywords:** hepatic artery dissection, hepatic artery thrombosis, isolated hepatic artery dissection, non surgical hepatic artery dissection, visceral artery dissection

## Abstract

Hepatic artery dissection means a tear in the wall of the hepatic artery, allowing blood to accumulate between arterial layers, leading to fatal or major sequelae, including ischemia and hepatic injury, pseudoaneurysm, or rupture. Spontaneous hepatic artery dissection is rare, especially in patients without any recent hepatobiliary surgery or trauma. It is a nonspecific and often subtle presentation that can delay diagnosis, leading to major/fatal consequences, including hepatic injury due to ischemia, pseudoaneurysm, and rupture leading to death, which highlights the need for clinical awareness.

We report on a 54-year-old man with hypertension, type 1 diabetes mellitus, hyperlipidemia, and a remote motor vehicle accident 12 years prior to our case presentation. He presented with acute epigastric pain. Computed tomography angiography (CTA) demonstrated an isolated dissection of the common and proper hepatic arteries without involvement of the aorta or celiac trunk. He was managed conservatively with anticoagulation and close follow-up. Repeat CTA at one month showed interval remodeling and partial recanalization of the hepatic artery. Although a few cases have been reported, they offer insights into risk factors, clinical presentation, and the range of management strategies. Our report underscores the importance of considering vascular causes in patients with unexplained upper abdominal pain. Early diagnosis and a tailored treatment plan can help prevent serious complications.

## Introduction

Dissection of a visceral artery in the absence of aortic involvement or recent intervention is rare. Among these, isolated hepatic artery dissection is particularly uncommon [1]. To better characterize this condition, we conducted a comprehensive review of all 1,238 published cases on PubMed from 1967 to 2024. Articles were excluded for lack of relevance, like hepatic artery aneurysm/pseudoaneurysm without dissection, or celiac artery dissection, for publications in languages other than English, and for publications where the full text was inaccessible. Twelve relevant cases, along with one from our institution, were included in the final analysis. Extracted data included demographics, risk factors, presentation, diagnostic methods, treatment, and outcomes.

Patients typically present in middle age (mean age approximately 60 years; range 38-82), and 77% (10 of 13) were male. The most common clinical presentation was abdominal pain (epigastric or right upper quadrant discomfort), observed in about 61-62% of cases. Hypertension or other atherosclerotic risk factors were present in ~38% of cases, and 54% had a prior surgical or endovascular procedure (e.g., chemotherapy catheter placement, cholecystectomy), suggesting an iatrogenic or post-traumatic etiology in some patients. Idiopathic (spontaneous) cases accounted for the remainder.

Computed tomography angiography (CTA) is the diagnostic modality of choice, allowing for the quick, noninvasive identification of dissection flaps, intramural hematoma, and any downstream complications such as pseudoaneurysm formation or visceral ischemia [2]. Catheter-based angiography may provide further anatomical detail in select cases, particularly when planning endovascular intervention, although its sensitivity can be reduced if the false lumen is thrombosed. 

Because isolated hepatic artery dissection is so rare, there are no universally established treatment algorithms. As such, management is typically based on the patient’s condition, including clinical stability, anatomical findings, and the presence or absence of complications. Consistent with broader management approaches for spontaneous isolated dissections of abdominal visceral arteries, conservative therapy is often the first-line strategy for hemodynamically stable and asymptomatic patients. This includes the use of antiplatelet or anticoagulant agents, blood pressure control, and close follow-up with serial imaging. When complications arise, such as evidence of compromised perfusion, expanding aneurysm/pseudoaneurysm, or persistent severe symptoms, endovascular interventions, like stenting or coil embolization, may be warranted. Surgical intervention, including bypass grafting or aneurysmorrhaphy, is generally reserved for cases involving rupture, progressive aneurysmal changes, or failure of less invasive treatments. These principles are supported by Shiraki et al., who analyzed clinical outcomes in patients with isolated splanchnic artery dissections and found that most could be managed conservatively, with only a minority requiring invasive procedures [3].

Here, we describe a case of spontaneous, isolated hepatic artery dissection in a patient without recent surgery or trauma, successfully managed with anticoagulation and close imaging surveillance. We also provide a focused literature review to elucidate prevailing trends in clinical presentation, diagnostic approaches, management strategies, and outcomes for this rare vascular entity.

## Case presentation

A 54-year-old Caucasian man with a past medical history of type 1 diabetes mellitus, hypertension, hyperlipidemia, benign prostatic hyperplasia, and a 30-pack-year history of tobacco use presented to the emergency department with the sudden onset of sharp epigastric pain radiating to the left chest. He denied nausea, vomiting, melena, hematochezia, or heartburn. His history was also notable for a remote motor vehicle accident 12 years prior and a provoked deep vein thrombosis (DVT) following a hip fracture that same year. 

On examination, vital signs were largely unremarkable except for elevated blood pressure at 168/92 mmHg. Laboratory testing, including complete blood count, comprehensive metabolic panel, and lactate level, was within normal limits. Inflammatory markers were notable for a mildly elevated C-reactive protein (CRP) of 3.7 mg/dl (normal range: <0.3 mg/dl). Electrocardiogram and cardiac biomarkers showed no evidence of acute coronary syndrome. 

**Figure 1:**
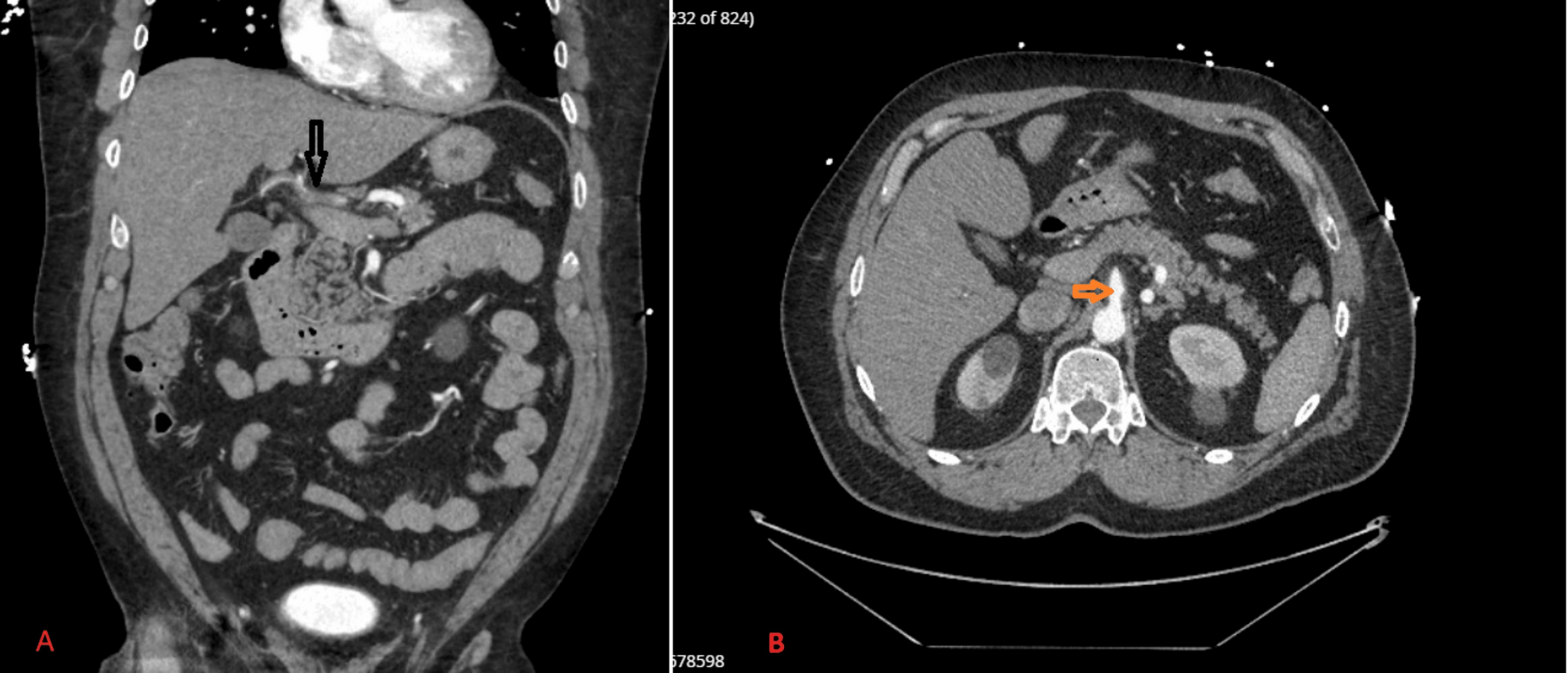
1A. Arterial phase CTA abdomen coronal view demonstrating a common hepatic artery dissection with intraluminal thrombus (black arrow). 1B. Arterial phase CTA abdomen axial view showing a patent celiac artery without extension of dissection (orange arrow). CTA: Computed Tomography Angiography

An initial CT abdomen/pelvis with contrast demonstrated inflammatory stranding surrounding the celiac axis and proximal common hepatic artery with concern for common hepatic artery thrombosis. CTA of the abdomen revealed a linear intimal flap in the common hepatic artery extending through the proper hepatic artery and into the distal right hepatic artery, consistent with arterial dissection. Perivascular inflammatory fat stranding was noted surrounding the celiac and common hepatic arteries, suggesting either an inflammatory reaction or, less likely, an underlying vasculitic process. No evidence of rupture, hepatic infarction, or involvement of the celiac trunk or superior mesenteric artery was observed (Figures 1A-1B). 

The patient was initially advised to be admitted for further workup, but he left against medical advice. He was discharged on aspirin 81 mg daily. Two days later, he returned to the hospital with worsening sharp epigastric pain radiating to the left chest. He remained hemodynamically stable. Given concern for ongoing dissection, a heparin infusion was initiated for anticoagulation.

An extensive autoimmune and coagulation workup was performed to evaluate for potential vasculitis or prothrombotic etiologies. Tests included antineutrophil cytoplasmic antibodies (ANCA), antinuclear antibody (ANA) with a reflex panel for extractable nuclear antigens, complement levels (C3 and C4), lupus anticoagulant, beta-2 glycoprotein antibodies, and anticardiolipin antibodies. Blood cultures were also obtained. All results were within normal limits or negative, except for an elevated b2-glycoprotein, which was not thought to be clinically significant in the absence of other anti-phospholipid antibodies, and no evidence of an inflammatory or autoimmune disorder was found, as shown in Table 1.

**Table 1:** Hypercoagulability and autoimmune workup Abbreviation: c-ANCA: Cytoplasmic Anti-Neutrophil Cytoplasmic Antibodies; Atypical p-ANCA: Atypical Perinuclear Anti-Neutrophil Cytoplasmic Antibodies; MPO AB: Myeloperoxidase Antibody; PR-3 AB: Proteinase-3 Antibody; P-ANCA: Perinuclear Anti-Neutrophil Cytoplasmic Antibodies; ANA: Antinuclear Antibody; AI: Antibody Index

Test	Result	Normal Range
c-ANCA	<1:20	<1:20
Atypical p-ANCA	<1:20	<1:20
MPO AB	<0.2 AI	<0.9 AI
PR-3 AB	<0.2 AI	<0.9 AI
P-ANCA	<1:20	<1:20
ANA with reflex panel	Negative	Negative
Complement C3	147 mg/dl	87-200 mg/dl
Complement C4	27 mg/dl	19-52 mg/dl
Lupus anticoagulant	38.0 seconds	0-43.5 seconds
Beta-2 glycoprotein IgG	1.3 U/ml	<7 U/ml (Negative), 7-10 (Equivocal), >10 (Positive)
Beta-2 glycoprotein IgA	14 U/ml	<7 U/ml (Negative), 7-10 (Equivocal), >10 (Positive)
Beta-2 glycoprotein IgM	<2.4 U/ml	<7 U/ml (Negative), 7-10 (Equivocal), >10 (Positive)
Anticardiolipin IgG	3.1 GPL	<10 GPL-U/ml (Negative), 10-40 (Weak Positive), >40 (Positive)
Anticardiolipin IgA	5.2 GPL	<10 GPL-U/ml (Negative), 10-40 (Weak Positive), >40 (Positive)
Anticardiolipin IgM	<9.0 GPL	<10 GPL-U/ml (Negative), 10-40 (Weak Positive), >40 (Positive)

Vascular surgery was consulted. In the absence of red-flag features, namely, hemodynamic instability, elevated liver function tests indicating hepatic ischemia, or worsening abdominal pain, a conservative management approach was recommended. The patient was transitioned from the heparin drip to apixaban 5 mg twice daily and discharged with outpatient vascular follow-up arranged.

A repeat CTA performed one month later demonstrated interval partial recanalization with arterial remodeling of the common hepatic and gastroduodenal arteries. A small residual dissection flap persisted at the bifurcation of the proper hepatic artery and gastroduodenal artery. At a three-month follow-up visit, the patient remained asymptomatic and had experienced no further complications.

## Discussion

Hepatic artery dissection (HAD) is an uncommon vascular pathology with diverse clinical presentations and an often multifactorial etiology. We reviewed the literature to better understand this condition and to summarize key trends among reported cases of isolated HAD. In our literature review of 13 cases (including the present report) (Table 2), the mean age was 60 years, and 10/13 (77%) of patients were male. Cardiovascular or metabolic risk factors, such as hypertension, diabetes mellitus, hyperlipidemia, smoking, or ischemic heart disease, were documented in 5 of 13 patients (38.5%). A prior history of surgery, trauma, or an endovascular procedure was noted in 7 of 13 cases (53.8%), including cholecystectomy, intra-arterial chemotherapy delivery, orthopedic surgery, and splenic artery embolization. The remaining cases were idiopathic (spontaneous). Other notable comorbidities included colorectal cancer with liver metastases in 3 of 13 cases (23.1%), fibromuscular dysplasia in 1 of 13 cases (7.7%), an autoimmune hemophilia-like disorder due to a factor XIII inhibitor in 1 of 13 cases (7.7%), and prior venous thromboembolism in 1 of 13 cases (7.7%).

**Table 2:** Demographic patterns, clinical presentations, risk factors, imaging findings, etiologies, management strategies, and outcomes in all reported cases of isolated hepatic artery dissection (12 cases from the literature and the current case) ASCVD: Atherosclerotic Cardiovascular Disease; DM” Diabetes Mellitus; HLD: Hyperlipidemia; HTN: Hypertension; RUQ: Right Upper Quadrant; CRC: Colorectal Cancer; CTA: Computed Tomography Angiography; DSA: Digital Subtraction Angiography; AP: Antiplatet; AC: Anticoagulant; Anti-HTN: Antihypertensive; GDA: Gastroduodenal Artery; FMD: Fibromuscular Dysplasia; MVA: Motor Vehicle Accident; N/R: Not Reported

First Author (Year)	Age, Sex	Initial Presentation	Hx of Smoking, ASCVD, DM, HLD, or HTN	Hx of Surgery, Trauma, or Endovascular Procedure	Other Medical History	Imaging Findings	Observation (Repeat Imaging)	Interventional Management	Medical Management (AP/AC/Anti-HTN)	Outcome
Carmody, 1994 [4]	60, M	Intra-arterial chemotherapy infusion (hepatic artery catheter)	N/R	Hepatic artery infusion for chemotherapy	CRC with hepatic metastasis	DSA: dissection flap in the hepatic artery	N/R (no initial observation; diagnosed during infusion)	Stent placement in the hepatic artery	N/R (not specified; antithrombotic not reported)	Multiple stent restenoses requiring repeat angioplasty; continued intra-arterial chemotherapy
Garcia, 1996 [5]	69, F	Acute abdominal pain with transaminitis (AST/ALT in thousands)	Myocardial infarction; HTN	N/R	On warfarin (for atrial fib or valve; not specified)	CTA & DSA: intimal flap in the hepatic artery	Follow-up arteriography at 2 months	None (conservative management)	Calcium channel blocker (nicardipine)	Complete resolution of dissection on the 2-month angiogram
Nakamura, 1998 [6]	51, M	Incidental finding on pre-op angiography (no symptoms)	N/R	N/R	CRC with hepatic metastasis (rectal primary)	DSA: dissection isolated to the left hepatic artery	N/R (immediate surgery due to concurrent metastasis)	Surgical reconstruction of the left hepatic artery (vein graft)	N/R (no specific medical therapy reported)	Patent hepatic artery anastomosis on postoperative arteriogram; alive 13 months post-op
Takayama, 2008 [7]	57, M	Incidental (no symptoms; found on imaging for another reason)	N/R	N/R	N/R	CT: focal intimal flap in hepatic artery; no thrombus	Periodic imaging follow-up (25 months)	None (observation only)	None reported	No false lumen expansion on follow-up at 25 months
Nishibe, 2013 [8]	60, M	Episodic epigastric pain	N/R	N/R	N/R	Pre-op imaging: proper hepatic artery aneurysmal dissection (size not stated)	N/R (proceeded to surgery)	Aneurysmorrhaphy and vein patch angioplasty (open repair)	N/R (no specific medical therapy reported)	Postoperative DSA showed a patent, proper hepatic artery; no complications at the 2-year follow-up
Tsuda, 2016 [9]	68, F	Sudden severe epigastric pain (initially thought to be pancreatitis)	N/R	Recent splenic artery embolization (for spontaneous splenic rupture)	Autoimmune acquired Factor XIII inhibitor (hemophilia); prior spontaneous bleeding	CTA: dissection flap in hepatic artery; perivascular stranding	Follow-up imaging (post-embolization monitoring)	None for hepatic artery (managed conservatively)	Supportive care (factor replacement; no specific anti-HTN or anticoagulant)	Hepatic artery dissection resolved spontaneously, with no ischemic complications
Su, 2016 [10]	43, F	Transient ischemic attack (amaurosis fugax), followed by severe RUQ pain and dizziness	HTN; history of TIA	Prior coronary and internal carotid artery dissections (peri-procedural); cholecystectomy	Fibromuscular dysplasia (multifocal)	CT: dissection flap in the common hepatic artery	Regular imaging follow-up (CT and MR angiography)	None (conservative management)	Dual antiplatelet therapy (aspirin + clopidogrel); ambulatory BP monitoring	Dissection flap persisted but was stable on repeat CT; no new symptoms
Bang, 2023 [11]	82, M	Epigastric pain, nausea, vomiting (initially suspected pancreatitis)	Ischemic heart disease; DM; dyslipidemia	N/R	N/R	CT: hepatic artery intimal flap (no celiac involvement)	Scheduled follow-up CT imaging	None (conservative management)	Anticoagulation (enoxaparin 60 mg BID)	Unchanged dissection on follow-up CT (no progression); clinically stable
Pinkerton, 1976 [12]	52, M	Episodic RUQ abdominal pain	N/R	N/R	Fibromuscular dysplasia (arterial wall pathology)	Angiography: dissecting aneurysm of the hepatic artery	N/R (immediate surgery due to aneurysm risk)	Surgical repair - aorto-hepatic bypass with 10-mm Dacron graft	N/R (not reported)	Full recovery; patent graft with no complications at 18-month follow-up
Bushkin, 1972 [13]	38, M	Blunt abdominal trauma (motor vehicle accident)	N/R	N/R (dissection occurred as a result of the trauma)	N/R	Surgical arteriogram: hepatic artery injury/dissection	N/R (injury recognized intraoperatively)	Surgical repair - aorto-hepatic bypass (graft to the hepatic artery)	N/R (not reported)	Recovered and discharged without complications
Crowhurst, 2011 [14]	65, F	Acute epigastric pain with pancreatitis (suspected biliary pancreatitis)	Myocardial infarction; hypercholesterolemia	Laparoscopic cholecystectomy (remote)	N/R	CT: dissection of the hepatic artery	Follow-up imaging at 8 months (CT scan)	None (observational management)	None (no specific medical therapy)	Complete resolution of dissection on follow-up imaging at 8 months
Brown, 2011 [15]	53 M	During arteriographic workup for liver metastasis, radioembolization (procedural complication)	N/R	N/R (no prior surgery; not a surgical candidate for tumor)	CRC with hepatic metastasis; on bevacizumab therapy	DSA: dissection of the common hepatic artery with pseudoaneurysm formation	Follow-up angiography at 4 weeks	Coil embolization of hepatic artery (performed 4 weeks after dissection)	Antiplatelet (clopidogrel for 4 weeks)	Pseudoaneurysm evident on repeat arteriogram; successfully excluded by coil embolization
Our Case, 2024	54, M	Acute epigastric pain radiating to the chest	HTN; DM; HLD; tobacco use	Remote motor vehicle accident (2012)	Provoked DVT (after hip fracture)	CTA: intimal flap in the common and proper hepatic arteries	Repeat CTA at 1 month: interval partial recanalization and remodeling	No invasive intervention (conservative management)	Anticoagulation (IV heparin in hospital; apixaban 5 mg BID on discharge)	Near-complete arterial healing on 1-month follow-up imaging (recanalization with remodeling); asymptomatic at 3 months

Acute abdominal pain (often epigastric or right upper quadrant (RUQ)) was the most frequent presenting symptom, occurring in approximately 8 of 13 cases (62%). Less common presentations included an asymptomatic or incidental finding on imaging in 1 out of 13 cases (~8%), neurological symptoms, such as dizziness or transient ischemic attack (~8%), or a presentation in the context of another condition (for example, concurrent acute pancreatitis in 1 out of 13 cases (~8%). Notably, our patient’s presentation with acute epigastric pain fits the most common pattern among reported cases. The case reported by Su et al. involved neurologic symptoms (transient ischemic attack (TIA)) preceding abdominal pain, suggesting fibromuscular dysplasia affecting multiple arteries [10]. In another case, hepatic artery dissection was discovered during an evaluation of pancreatitis (Crowhurst et al.) and was likely secondary to the inflammatory process [14]. Two cases were entirely asymptomatic, with the dissection detected incidentally on imaging (e.g., during evaluation for metastatic disease or unrelated symptoms) [6,7]. These differences emphasize the importance of maintaining clinical suspicion in patients with risk factors or unusual presentations.

CTA has been the cornerstone for diagnosis in recent cases, providing noninvasive visualization of the intimal flap, any intramural hematoma, and an assessment for organ compromise. In older reports, conventional angiography (digital subtraction angiography) was often used to confirm the diagnosis [1,15]. In our patient, CTA readily identified the dissection and guided a conservative management plan. Catheter angiography may be helpful for cases where endovascular intervention is being considered or when the diagnosis is unclear on noninvasive imaging. Across the cases, no instance of misdiagnosis was reported after dedicated vascular imaging was performed, although initial presentations were often misattributed to more common conditions (e.g., pancreatitis, peptic ulcer disease, musculoskeletal pain).

The management of isolated HAD was heterogeneous and largely tailored to each patient’s clinical stability and the extent of the dissection. Overall, a conservative approach was favored in the majority of reported cases, especially in hemodynamically stable patients without signs of organ ischemia. For such patients, management typically involves blood pressure control and antithrombotic therapy with close imaging surveillance. For example, Garcia et al. reported management with nicardipine (a calcium channel blocker) and observed complete spontaneous healing of the dissection [5]. Similarly, Su et al. managed the fibromuscular dysplasia-related dissection with dual antiplatelet therapy and careful follow-up, avoiding invasive procedures [10]. The current patient was managed with a similar approach, as the patient’s stable condition allowed for successful management with anticoagulation and observation, which showed arterial remodeling on follow-up imaging.

In contrast, invasive interventions were reserved for patients who developed complications or had high-risk anatomy. Surgical interventions, including bypass grafting or arterial reconstruction, were performed in select cases with complications such as aneurysmal dilation of the dissected artery, evidence of rupture, or failure of conservative management [6,8,12,13]. Examples include Nakamura et al., who performed a saphenous vein graft bypass of the left hepatic artery during liver resection [6], and Nishibe et al., who repaired a dissecting hepatic aneurysm with aneurysmorrhaphy and patch angioplasty [8]. Similarly, early case reports by Pinkerton and by Bushkin from the 1970s involved open surgical repair, given the limited alternatives at that time and the presence of fibrodysplastic aneurysm or traumatic dissection, respectively [1]. Endovascular therapies, such as stent placement or coil embolization, were utilized in a few instances [4,15]. Carmody et al. deployed an intravascular stent to maintain hepatic artery patency during chemotherapy infusion [1], although that patient later required multiple re-interventions for stent restenosis. Brown et al. reported using coil embolization to treat a pseudoaneurysm that developed at the site of dissection in a patient on bevacizumab [15]. Adjunctive medical therapy was implemented in nearly half of the cases, encompassing antiplatelet agents, anticoagulants, and antihypertensive medications as appropriate [5,10,11,15]. For instance, anticoagulation with heparin or low-molecular-weight heparin was chosen in two recent cases by Bang et al. to prevent thrombus propagation in the dissected artery [11], and antiplatelet therapy was favored in the fibromuscular dysplasia (FMD)-related dissection by Su et al. to mitigate the risk of further endothelial injury [10]. No universal treatment guideline exists, and the approach should be tailored to each patient.

Outcomes were favorable in most instances, with no mortality reported among the 13 cases reviewed. Imaging demonstrated complete or near-complete resolution of the dissection in approximately 8 of 13 cases (62%) on follow-up (typically within 2-12 months). About 2 of 13 cases (15%) had persistent but stable dissections on follow-up imaging, without clinical deterioration. Approximately 2 of 13 cases (15%) experienced a complication related to the dissection or its treatment, such as stent restenosis or pseudoaneurysm formation requiring further intervention [15]. The case by Carmody et al. was complicated by recurrent stent occlusions that necessitated multiple angioplasties, and the case by Brown et al. developed a pseudoaneurysm that was successfully managed with coil embolization. Despite these complications, all patients were alive at last follow-up, and the majority had either recovered fully or had stable disease without significant long-term sequelae. Our patient likewise had a favorable outcome, with imaging evidence of vascular remodeling, and remained symptom-free at three months. These cases indicate that, in properly selected patients (those without rupture or organ compromise), medical management alone can lead to spontaneous healing of the dissection. Invasive treatment may be needed only if conservative measures fail. Early recognition and an appropriate, case-specific management plan are critical to prevent catastrophic events such as hepatic infarction or uncontrolled hemorrhage.

For hemodynamically stable patients without evidence of rupture or end-organ ischemia, a noninvasive approach is reasonable with anticoagulation or antiplatelet therapy (depending on the clinical context) and strict blood pressure control. Close surveillance with follow-up imaging is essential to ensure the dissection is not progressing. Invasive intervention should be reserved for patients who show clinical deterioration or high-risk anatomical features on imaging (e.g., an expanding false lumen, branch vessel occlusion, or impending rupture). This approach is consistent with strategies used for other visceral artery dissections and is supported by the overall positive outcomes observed in the limited literature to date.

Nevertheless, there is potential for reporting bias, as most available data are derived from published case reports, which may overrepresent favorable outcomes. Additionally, follow-up duration and outcome reporting were heterogeneous, with widely varying follow-up intervals across cases, limiting interpretation of long-term outcomes.

## Conclusions

Isolated hepatic artery dissection, though rare, should be considered in the differential diagnosis of patients presenting with unexplained acute upper abdominal pain, especially in those with relevant risk factors or history. Timely recognition via CTA is essential for diagnosis. In the absence of rupture, significant aneurysmal dilation, or organ ischemia, conservative management with careful blood pressure control and anticoagulation or antiplatelet therapy has been associated with good outcomes in reported cases, including this patient. Invasive interventions should be individualized and are typically considered only in the setting of complications or high-risk features. More published cases are needed to establish more definitive management guidelines for this unusual condition.
